# Assessing the Effect of Abstinent Duration on Brain Function in Heroin‐Dependent Individuals During Protracted Abstinence: A Resting‐State fMRI Study

**DOI:** 10.1111/adb.70097

**Published:** 2025-12-05

**Authors:** Xin Li, Wei Li, Jiajie Chen, Long Jin, Zhidong Wang, Liyang Dang, Wei Wang, Yue Qin, Qiang Li

**Affiliations:** ^1^ Department of Radiology Tangdu Hospital, Fourth Military Medical University Xi'an Shaanxi China; ^2^ Department of Radiology Xi'an Daxing Hospital Xi'an Shaanxi China; ^3^ Department of Nuclear Medicine Tangdu Hospital, Fourth Military Medical University Xi'an Shaanxi China

**Keywords:** abstinence, heroin‐dependent individuals, protracted abstinence, resting‐state functional magnetic resonance imaging

## Abstract

Protracted abstinence (PA) is the commonly implemented treatment of heroin‐dependent individuals (HDIs) in China. However, the effect of abstinence duration on the brain function of HDIs during PA using resting‐state functional magnetic resonance imaging (fMRI) remains unclear. Fourteen HDIs who had finished PA for about 6 months (PA6), 16 HDIs who had completed PA for about 11 months (PA11) and 15 demographically matched healthy controls (HC) underwent this fMRI study. We analysed the difference in amplitude of low‐frequency fluctuation (ALFF) values among the three groups. Then we analysed the difference in functional connectivity (FC) based on the differential regions of ALFF. Additionally, we examined the relationship between FC of differential brain regions and abstinence duration. The differences in ALFF among the three groups were found to be significant in the bilateral putamen and left inferior parietal lobule (single voxel *p* < 0.001, cluster level *p* < 0.05 and GRF‐corrected). Compared with the PA6 group, the PA11 group showed lower ALFF values of the differential regions with a tendency toward the HC group. Meanwhile, the PA11 group showed lower FC between the left putamen and left insula, between the right putamen and left insula and between the left inferior parietal lobule and bilateral inferior frontal gyrus (IFG), but higher FC between the left putamen and left inferior temporal gyrus. The above FC of HDIs negatively correlated with the abstinence duration, except for the left putamen–inferior temporal gyrus FC. The prolonged abstinence duration may be useful to restore the impaired brain function of HDIs to some extent, although more data are needed to validate this in future studies.

## Graphical abstract

This study found that a prolonged abstinence was associated with decreased functional activity in the bilateral putamen and inferior parietal lobule, with trends toward those of healthy controls. Additionally, partial functional connectivity networks demonstrated a recovery tendency after long‐term abstinence. These results indicated that longer abstinence durations in heroin‐dependent individuals were associated with recovery of brain activity patterns.

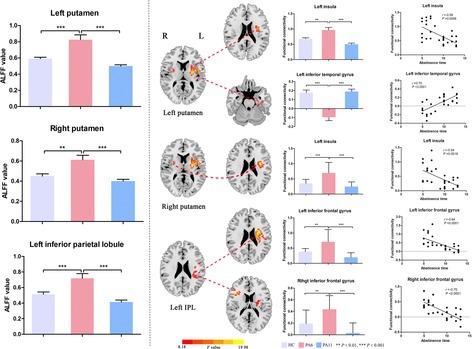

## 1 Introduction

Heroin addiction is a severe form of opioid use disorder, characterized as a chronic relapsing condition with a high propensity for physical dependence and compulsive drug‐seeking behaviour [1]. Abstinence has long been a cornerstone in the treatment of substance use disorders [2]. In China, protracted abstinence (PA) is an administrative intervention and drug rehabilitation measure stipulated in the Compulsory Drug Rehabilitation Law. This approach aims to enhance the efficacy of addiction treatment and reduce the relapse rate of heroin addiction through a prolonged abstinence period, given that short‐term abstinence has shown limited success in preventing recurrence [3, 4]. Consequently, the law was revised to extend the abstinence duration to 1–3 years. However, the effect of long‐term abstinence on the brain function of HDIs remains unclear.

Neuroimaging research has made substantial contributions to elucidating post‐addiction neuroanatomical and functional brain alterations. In alcohol use disorder patients undergoing long‐term abstinence, studies have reported increased glutamate receptor activity [5] and restoration of cerebral blood flow [6]. Emerging evidence indicates that prolonged abstinence is associated with partial recovery of brain structure and function in individuals with substance use disorders [7, 8, 9]. However, whether extending abstinence duration promotes brain functional recovery in HDIs remains unclear. Our previous study [10] demonstrated that during short‐term abstinence, HDIs exhibited abnormally enhanced cue‐induced brain responses in the default mode network (DMN), salience network (SN) and dopaminergic regions—findings suggesting persistent vulnerability to relapse despite brief abstinence. Shen et al. [11] used diffusion tensor imaging (DTI) to examine brain structure across different abstinence phases in HDIs, revealing disrupted corpus callosum integrity during early abstinence that showed signs of recovery after prolonged abstinence. Additionally, a resting‐state functional connectivity (FC) study in stimulant use disorder found that longer abstinence durations were associated with reduced FC in decision‐making and reward‐processing networks among successful abstainers [12]. Thus, applying resting‐state functional magnetic resonance imaging (fMRI) to explore how abstinence duration impacts brain function in HDIs during PA is essential. Such research will advance our understanding of the neurobiological mechanisms underlying long‐term abstinence on heroin addiction.

Resting‐state functional magnetic resonance imaging (rs‐fMRI) uses low‐frequency blood oxygen level‐dependent (BOLD) signal fluctuations during rest to characterize functional connections in normal and pathological brains across clinical contexts [13, 14]. This technique employs two primary approaches for neural network identification: functional segregation (e.g., amplitude of low‐frequency fluctuations, ALFF) and functional integration (seed‐based FC analysis) [15]. ALFF is considered a proxy for regional neural activity intensity [16], while seed‐based connectivity reflects signal synchronization between a predefined seed region and other brain areas—an analysis that often incorporates ALFF‐derived metrics [15]. Specifically, seed‐based FC analysis uses temporal BOLD signal fluctuations at low frequencies to quantify the degree of synchronization between regions of interest (ROIs) and the rest of the brain [17]. Previous research has used resting‐state FC to decode brain circuitry organization and evaluate neurophysiological alterations in substance use disorders [18, 19].

In this fMRI study, we enrolled two groups of HDIs with different abstinence durations and a healthy control (HC) group. We analysed the differences in ALFF and FC induced by prolonged abstinence in HDIs. We hypothesized that longer abstinence would facilitate better recovery of brain function in HDIs.

## 2 Materials and Methods

### 2.1 Subjects

Fourteen HDIs during PA for 6 months (PA6) (11 males, 3 females; aged from 26‐ to 43‐years old), 16 HDIs during PA for 11 months (PA11) (13 males, 3 females; aged from 27‐ to 46‐years old) and 15 demographically matched HC (13 males, 2 females; aged from 30‐ to 43‐years old) were included in this fMRI study. Detailed demographic characteristics and medication histories of all participants were presented in Table 1. Thirty abstinent HDIs were recruited from the Drug Rehabilitation Center in Lantian, Xi'an. During the abstinence period, they only received educational guidance and physical exercise without any pharmacologic assistance. The inclusion criteria for the HDIs were as follows: (1) meeting the diagnostic criteria of DSM‐V for opioid dependence; (2) right‐handedness; (3) abstinence durations of approximately 6 months (PA6) and 11 months (PA11); (4) no history of methadone maintenance treatment; and (5) negative urine test on the day of the experiment. All the participants would be excluded if they had a history of (1) history of mental illness or neurological, infectious diseases (e.g., AIDS); (2) head trauma, colour blindness; (3) contraindication to magnetic resonance examination. The study was approved by the Ethics Committee of Tangdu Hospital. All participants provided written informed consent before the experiment.

**TABLE 1:** Demographic information and medication histories of participants.

	PA11	PA6	HC	*F*/*t*/χ^2^	*p*
Number of cases	16	14	15	/	/
Age (years)^a^	33.69 ± 6.35	36.29 ± 5.55	37.93 ± 3.88	0.35	0.84
Sex (male/female)^b^	13/3	11/3	13/2	1.71	0.43
Education (years)^a^	9.56 ± 2.50	10.85 ± 2.93	10.20 ± 3.00	0.79	0.46
Smoking duration (years)^a^	16.56 ± 6.73	15.93 ± 6.37	17.60 ± 1.48	0.26	0.77
Daily dosage of heroin (g)^c^	0.73 ± 0.47	0.65 ± 0.52	/	−0.06	0.95
Duration of heroin use (months)^c^	88.69 ± 50.51	87.86 ± 52.64	/	0.42	0.68
Total dosage of heroin use (g)^c^	1907.34 ± 529.48	2282.43 ± 547.23	/	−0.45	0.65
Abstinent duration (months)^c^	11.13 ± 1.41	6.07 ± 0.24	/	11.44	0

Abbreviations: HC, healthy controls; PA6, protracted abstinence for about 6 months; PA11, protracted abstinence for about 11 months.

^a^ One‐way analysis of covariance (ANCOVA).

^b^ Chi‐square test.

^c^ Independent sample *t* test.

### 2.2 Brain Imaging Methodology and Data Analysis

#### 2.2.1 MRI Scanning

Before the MRI scan, participants were prohibited from consuming alcohol, caffeine or any other drugs/medications for 12 h. The MRI data were acquired using a GE Signa Excite HD 3.0‐T MR scanner (Milwaukee, WI, USA) with an eight‐channel head coil. The subjects were instructed to lie supine, remain motionless, avoid focused thinking and stay awake during the scan, and their adherence to these requirements was confirmed via a brief structured interview immediately after the scanning procedure. The scan range covered the whole brain. Conventional T2‐weighted imaging (T2WI) sequence of the brain was routinely employed for detecting intracranial lesions. Resting‐state fMRI data were collected using an echo planar imaging sequence with the following parameters (TR = 2000 ms, TE = 30 ms, flip angle = 90°, imaging matrix = 64 × 64, field of view = 256 × 256 mm^2^, slice thickness = 4 mm, gap = 0 mm and number of slices = 32). At each time point, 32 axial images were acquired; a total of 150 time points were collected over a 5‐min scan duration.

#### 2.2.2 Data Preprocessing

The fMRI images were preprocessed using Matlab, DPABI and SPM12 software. The fMRI data were preprocessed including the following steps: slice timing, head‐motion correction, spatial normalization to a template at the Montreal Neurological Institute space, spatial smoothing with a 6‐mm Gaussian kernel, removal of linear detrend, nuisance signal regression (including signals of cerebrospinal fluid [CSF], white matter and 24 head‐motion parameters) and temporal bandpass filtering (0.01–0.08 Hz). The ALFF values were calculated. The difference in ALFF values among the above HC, PA6 and PA11 groups was analysed using one‐way ANOVA.

#### 2.2.3 FC Analysis

The seed‐based FC method was used to assess the resting‐state properties of the three groups. The spherical ‘seed’ regions were defined based on the differential brain regions of ALFF value among the three groups (diameter = 6 mm) [20, 21]. The FC was calculated and converted to Z value using Fisher's z‐transformation and then submitted to the one‐way ANOVA among the PA6, PA11 and HC groups. The differences in FC among the three groups were analysed. The Pearson correlation between the FC strength of differential brain regions and abstinent duration was analysed.

A *p*‐value < 0.05 was considered statistically significant between groups.

## 3 Results

### 3.1 Demographic Characteristics and Clinical Data

The HC, PA6 and PA11 groups showed no significant differences in age, sex, education years or smoking history (all *p* > 0.05). Similarly, the two HDI subgroups (PA6 and PA11) also did not differ significantly in daily heroin dose, duration of heroin use or total dosage of heroin use (Table 1).

### 3.2 Difference in ALFF

**FIGURE 1:**
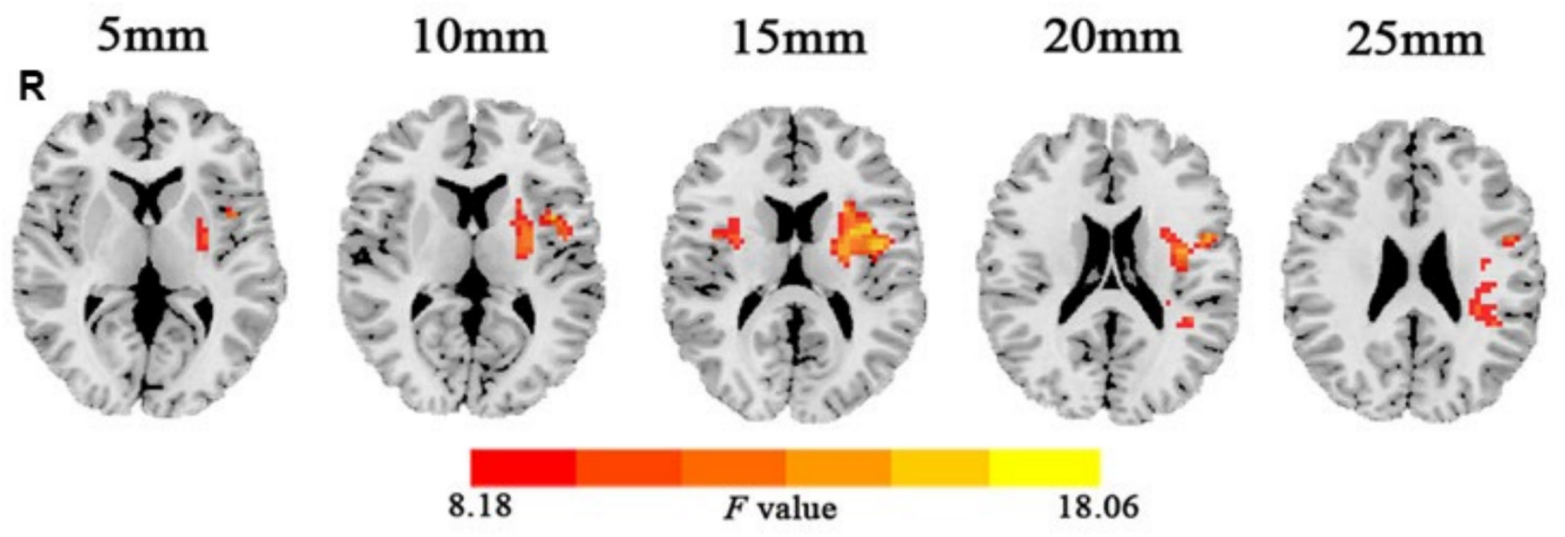
ALFF differences among three groups (single voxel *p* < 0.001, cluster *p* < 0.05 and GRF‐corrected): bilateral putamen and left inferior parietal lobule.

**FIGURE 2:**
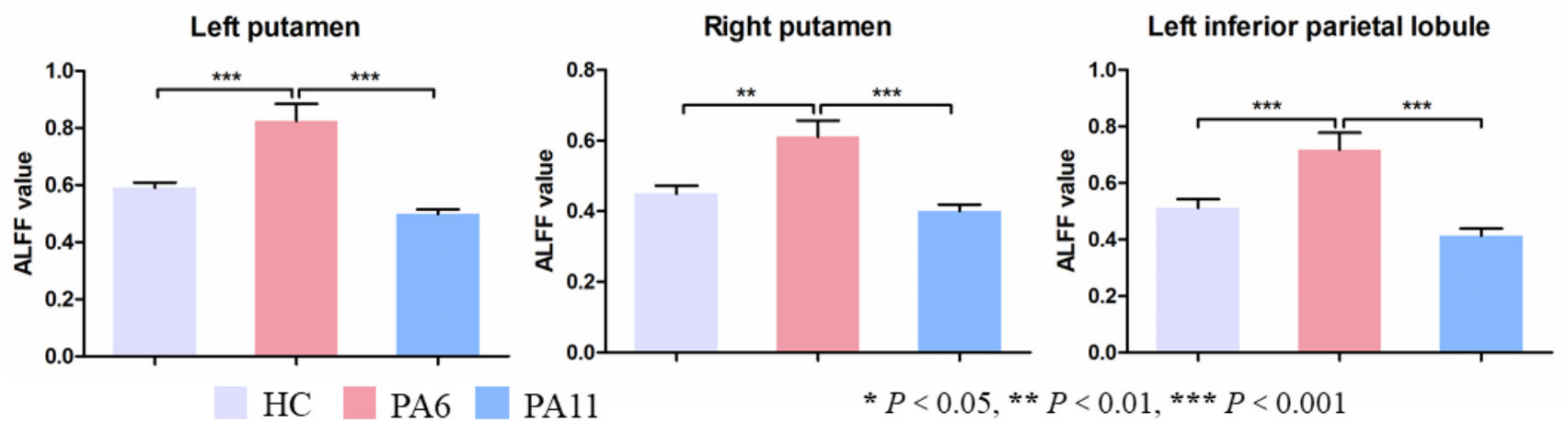
Post hoc results of the difference in ALFF value among the three groups. HC = healthy controls, PA6 = protracted abstinence for about 6 months, PA11 = protracted abstinence for about 11 months.

Among the PA6, PA11 and HC groups, significant differences in ALFF value were observed in the bilateral putamen and left inferior parietal lobule (Table 2, Figure 1) (single voxel *p* < 0.001, cluster level *p* < 0.05 and GRF‐corrected). Post hoc tests revealed that compared with PA6, both the PA11 and HC groups showed significantly decreased ALFF values in the bilateral putamen and left inferior parietal lobule (Figure 2). No statistically significant differences in ALFF values were observed in the above brain regions between the PA11 and HC groups (*p >* 0.05).

**TABLE 2:** Differences in the ALFF value among three groups (pixel *p* < 0.001, cluster *p* < 0.05 and GRF‐corrected).

Brain regions	BA	MNI			*F* value	Clusters
		x	y	z		
Left putamen	—	−27	−9	12	18.06	45
Right putamen	48_R	30	−3	12	11.56	32
Left inferior parietal lobule	48_L	−33	−36	24	11.45	34

Abbreviations: L, left; MNI, Montreal Neurological Institute; R, right.

### 3.3 FC Results and the Correlation Between FC Alteration and Abstinence Duration

#### 3.3.1 Using the Left Putamen as the Region of Interest (ROI)

**FIGURE 3:**
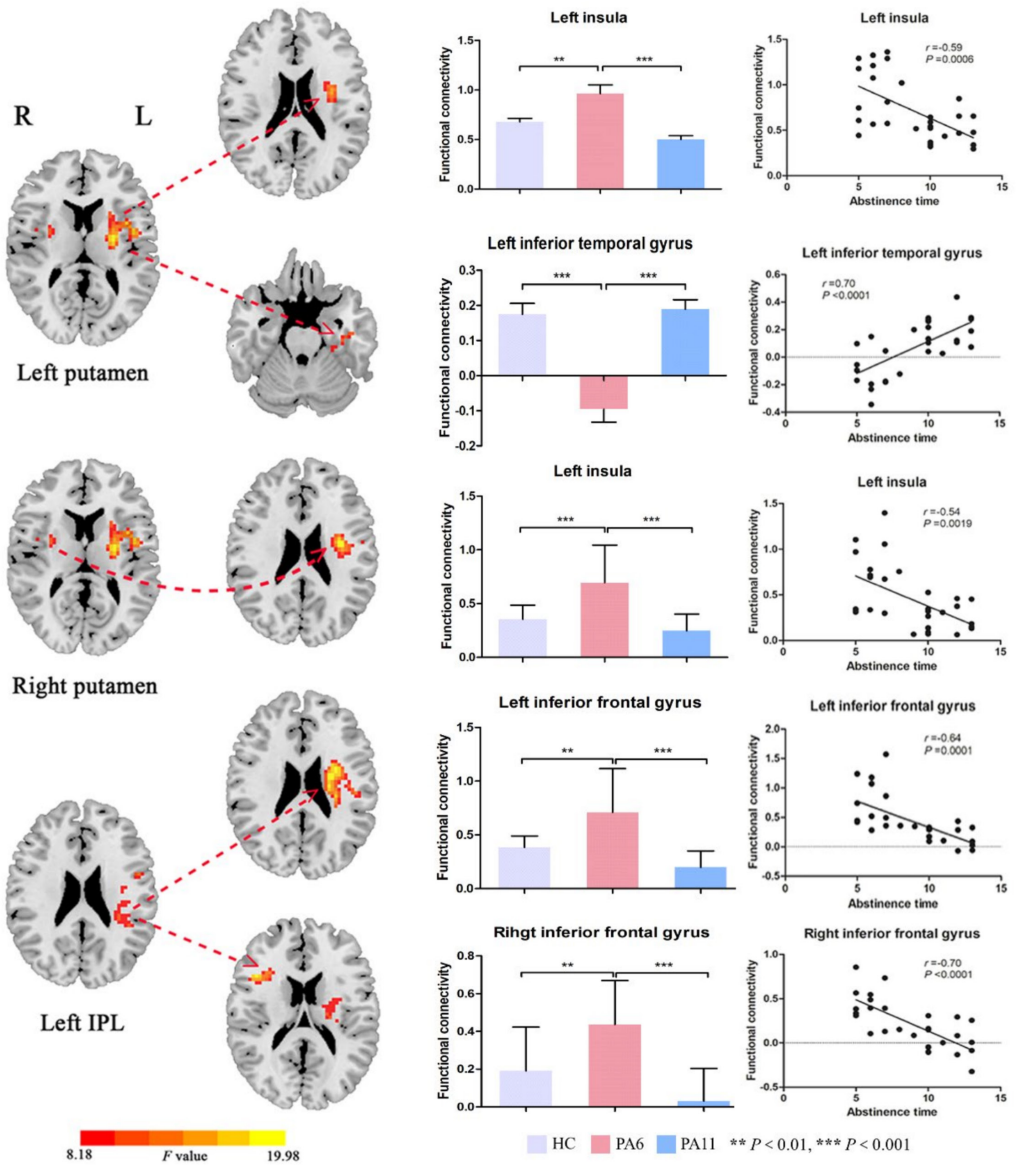
Seed‐based functional connectivity and the correlation between FC and abstinent duration. The seed regions included the bilateral putamen and left inferior parietal lobule (IPL). HC = healthy controls, PA6 = protracted abstinence for about 6 months, PA11 = protracted abstinence for about 11 months.

Using the left putamen as the region of interest (ROI), significant intergroup differences in FC were identified in the left insula and left inferior temporal gyrus (ITG) across the three groups (single voxel *p* < 0.001, cluster level *p* < 0.05 and GRF‐corrected) (Table 3). Compared with PA6, both HC and PA11 groups exhibited decreased FC between the left putamen and left insula, but enhanced FC between the left putamen and left ITG. Abstinence duration showed a significant negative correlation with left putamen–left insula connectivity (*r* = −0.59, *p* = 0.0006) and a positive correlation with left putamen–left ITG connectivity (*r* = 0.70, *p* < 0.0001) (Figure 3).

**TABLE 3:** Differences in the functional connectivity of the regions with altered ALFF value among three groups (pixel *p* < 0.001, cluster *p* < 0.05 and GRF‐corrected).

Seed	Brain regions	BA	MNI			*F* value	Clusters
			x	y	z		
Left putamen	Left ITG	20_L	−45	−15	−24	11.02	38
	Left insula	48_L	−33	−3	21	14.86	103
Right putamen	Left insula	48_L	−33	−6	24	19.98	83
Left IPL	Left IFG	48_L	−30	0	24	14.41	34
	Right IFG	48_R	42	18	18	14.04	154

Abbreviations: IFG, inferior frontal gyrus; IPL, inferior parietal lobule; ITG, inferior temporal gyrus; MNI, Montreal Neurological Institute.

#### 3.3.2 Using the Right Putamen as the ROI

Using the right putamen as the ROI, significant intergroup differences in FC were observed in the left insula across the three groups (single voxel *p* < 0.001, cluster level *p* < 0.05 and GRF‐corrected) (Table 3). Compared with PA6, both HC and PA11 groups exhibited decreased FC between the right putamen and left insula. In HDIs, right putamen–left insula connectivity showed a significant negative correlation with abstinence duration (*r* = −0.54, *p* = 0.0019) (Figure 3).

#### 3.3.3 Using the Left Inferior Parietal Lobule as the ROI

Using the left inferior parietal lobule as the ROI, significant intergroup differences in FC were identified in the bilateral IFG across the three groups (single voxel *p* < 0.001, cluster level *p* < 0.05 and GRF‐corrected) (Table 3). Compared with PA6, both HC and PA11 groups exhibited decreased FC between the left inferior parietal lobule and bilateral IFG. Connectivity between the left inferior parietal lobule and left IFG, as well as between the left inferior parietal lobule and right IFG, showed significant negative correlations with abstinence duration (*r* = −0.64, *p* = 0.0001; *r* = −0.70, *p* < 0.0001) (Figure 3).

## 4 Discussion

Our findings partially supported the hypothesis, demonstrating that (1) HDIs in the PA11 group exhibited reduced local spontaneous brain activity in bilateral putamen and left inferior parietal lobule compared to the PA6 group, tending toward levels observed in the HC group; (2) compared to the PA6 group, the PA11 group exhibited bidirectional alterations in FC, with its connection pattern approaching those of the HC group. Additionally, brain function trended toward recovery as the abstinence duration prolonged. These results indicated that longer abstinence durations in HDIs were associated with progressive recovery of brain activity patterns, shifting toward HC‐like profiles. This study contributed to the neuroimaging literature by evaluating how abstinence duration modulated brain function in HDIs.

In HDIs, long‐term abstinence was associated with reduced local neural activity in the putamen and decreased FC between the putamen and insula, with the latter showing a negative correlation with abstinence duration. The putamen, as part of the striatum, is central to addiction‐related neural mechanisms [22]. Together with the ventral striatum (e.g., nucleus accumbens), it forms the dopaminergic reward circuit, which mediates addictive reward processing and habitual behaviour [23, 24]. Positron emission tomography (PET) studies have shown that prolonged abstinence leads to significantly reduced dopamine transporter (DAT) uptake in the striatum, indicating restoration of dopaminergic neuronal function following long‐term abstinence [25]. The insula serves as a core node of the SN, playing a pivotal role in higher‐order cognitive control and attentional processing [26]. Previous research has demonstrated that heroin‐related cues elicit decreased insular activity after prolonged abstinence [27]. Notably, Naqvi et al. found that smokers with insular damage showed higher rates of successful quitting and lower relapse propensity [28], highlighting the insula's role in addiction remission. In drug addiction and other neuropsychiatric disorders, both the mesolimbic dopamine pathway and the cortical SN showed dysregulations. PET and resting‐state fMRI studies have shown that decreased dopaminergic transmission is associated with disrupted connectivity in the SN [29]. These findings indicated that the SN and mesolimbic dopamine system have close functional interactions, which played a critical role in drug addiction recovery. However, longitudinal studies are required to characterize their dynamic changes across different abstinence phases and clarify their mechanistic roles in neuroplasticity.

The ITG is part of the DMN, playing a key role in vision, language, multisensory processing and behavioural control [30, 31, 32, 33]. Prior research has linked reduced language learning and memory in opioid‐dependent individuals to the left temporal region [34]. One study showed that heroin relapsers had decreased FC of the left ITG with the DMN compared to abstainers [35]. Additionally, heroin intake has been associated with reduced regional cerebral blood flow in the ITG, a potential contributor to brain damage in addiction [31]. An event‐related fMRI study found that prolonged abstinence attenuated cue‐induced responses in visuospatial attention regions like the ITG [10]. Our study revealed significantly enhanced FC between the left putamen and left ITG in both PA11 and HC groups versus PA6, indicating gradual restoration toward normal with extended abstinence. This supports the hypothesis that longer abstinence promotes functional recovery in these neural pathways.

This study revealed that prolonged abstinence promoted normalization of frontoparietal regional neural activity and FC in HDIs toward HC levels. The inferior parietal lobule influences substance addiction maintenance and relapse through mechanisms like attentional regulation [36], inhibitory control [37] and risk decision‐making [38]. The IFG, a prefrontal cortex hub, is critical for inhibitory control and higher‐order cognitive functions [39, 40]. Neurocognitive research has implicated the IFG in the cognitive regulation of drug cravings [41, 42], with studies showing that opioid‐dependent individuals abstinent for at least 1 year exhibit comparable cognitive performance to HCs [43]. An 8‐month longitudinal study of heroin addiction observed partial recovery of frontal lobe structure and associated neural circuits [44]. Our cross‐sectional findings demonstrated recovery of frontoparietal FC after 11 months of abstinence, suggesting that alterations in frontoparietal connectivity may serve as a potential neuroimaging biomarker for functional recovery in HDIs. Longitudinal studies are needed to validate these findings.

This study has several limitations: Firstly, the sample size was small, with a low proportion of female participants. Future studies should enrol a larger sample size and include more female HDIs. Secondly, due to the challenge of recruiting participants with PA, this was a cross‐sectional study. The observed association between brain functional changes and abstinence duration cannot be directly interpreted as a causal relationship. Future studies should adopt longitudinal designs, conducting repeated measurements on the same group of heroin‐dependent individuals at multiple time points to more reliably elucidate the dynamic causal pathways through which abstinence duration promotes functional brain recovery. It lacked follow‐up data on relapse and other abstinence‐related cognitive/behavioural indicators, so conclusions about brain activity and connectivity changes with prolonged abstinence were inferred from fMRI trends. Longitudinal research is needed to track neuroplastic changes over extended abstinence periods. Finally, a common limitation in resting‐state fMRI studies is the inability to ensure participants avoid special thinking during scanning, which may introduce confounds to FC measurements.

In conclusion, our findings show that prolonged abstinence is associated with decreased functional activity in the bilateral putamen and inferior parietal lobule, with values approaching those of HC. Additionally, partial FC networks demonstrated a recovery trend after long‐term abstinence. Future research, particularly longitudinal studies, is needed to validate these results and identify potential biomarkers for the treatment of heroin addiction.

## Author Contributions

**Xin Li:** writing – original draft, data curation. **Wei Li:** writing – original draft, data curation. **Jiajie Chen:** formal analysis, writing – original draft. **Long Jin:** writing – review and editing. **Zhidong Wang:** data curation. **Liyang Dang:** writing – review and editing. **Wei Wang:** supervision, writing – review and editing. **Yue Qin:** conceptualization, investigation, writing – review and editing. **Qiang Li:** conceptualization, methodology, formal analysis, visualization.

## Funding

This study was supported by The Discipline Boosting Plan Tangdu Hospital (2025JCRH041).

## Conflicts of Interest

The authors declare no conflicts of interest.

## Data Availability

The data that support the findings of this study are available on request from the corresponding author. The data are not publicly available due to privacy or ethical restrictions.
